# Breathprints for Breast Cancer: Evaluating a Non-Invasive Approach to BI-RADS 4 Risk Stratification in a Preliminary Study

**DOI:** 10.3390/cancers18020226

**Published:** 2026-01-11

**Authors:** Ashok Prabhu Masilamani, Jayden K. Hooper, Md Hafizur Rahman, Romy Philip, Palash Kaushik, Geoffrey Graham, Helene Yockell-Lelievre, Mojtaba Khomami Abadi, Sarkis H. Meterissian

**Affiliations:** 1 Noze, 4920 Pl. Olivia, Saint-Laurent, QC H4R 2Z8, Canada; 2 Department of Surgery and Oncology, McGill University, Montreal, QC H4A 3J1, Canada

**Keywords:** breast cancer, BI-RADS 4, breath analysis, volatile organic compounds (VOCs), digital olfaction (electronic nose), chemiresistive sensor array, machine learning, multi-modal fusion, autoencoder, risk stratification, rule-out diagnostics

## Abstract

**Background/Objectives**: Breast cancer is the most common malignancy among women, and early detection is critical for improving outcomes. The Breast Imaging Reporting and Data System (BI-RADS) standardizes reporting, but the BI-RADS 4 category presents a major challenge, with malignancy risk ranging from 2% to 95%. Consequently, most women in this category undergo biopsies that ultimately prove unnecessary. This study evaluated whether exhaled breath analysis could distinguish malignant from benign findings in BI-RADS 4 patients. **Methods**: Participants referred to the McGill University Health Centre Breast Center with BI-RADS 3–5 findings provided multiple breath specimens. Breathprints were captured using an electronic nose (eNose) powered breathalyzer, and diagnoses were confirmed by imaging and pathology. An autoencoder-based model fused the breath data with BI-RADS scores to predict malignancy. Model performance was assessed using repeated cross-validation with ensemble voting, prioritizing sensitivity to minimize false negatives. **Results**: The breath specimens of eighty-five participants, including sixty-eight patients with biopsy-confirmed benign lesions and seventeen patients with biopsy-confirmed breast cancer within the BI-RADS 4 cohort were analyzed. The model achieved a mean sensitivity of 88%, specificity of 75%, and a negative predictive value (NPV) of 97%. Results were consistent across BI-RADS 4 subcategories, with particularly strong sensitivity in higher-risk groups. **Conclusions**: This proof-of-concept study shows that exhaled breath analysis can reliably differentiate malignant from benign findings in BI-RADS 4 patients. With its high negative predictive value, this approach may serve as a non-invasive rule-out tool to reduce unnecessary biopsies, lessen patient burden, and improve diagnostic decision-making. Larger, multi-center studies are warranted.

## Simple Summary

Breast cancer screening often identifies findings that are suspicious but uncertain, especially those labeled as BI-RADS 4. While doctors usually recommend a biopsy for these cases, most turn out to be benign, meaning many women go through an invasive procedure unnecessarily. This study explored whether a simple breath test could help better identify high-risk patients. By analyzing patterns of natural chemicals in exhaled breath, we trained a computer model to distinguish between cancerous and non-cancerous findings. The model was able to correctly identify most cancers while also giving strong reassurance when no cancer was present. These results suggest that a breath test could be used alongside mammography to provide patients and doctors with clearer information. If confirmed in larger studies, this approach could spare many women from unnecessary biopsies, lower healthcare costs, and improve trust in breast cancer screening.

## 1. Introduction

Breast cancer remains a major global health challenge, and early and accurate diagnosis is crucial for improving survival [1,2,3]. The current standard for detection relies on screening mammography and a standardized classification system developed by the American College of Radiology called Breast Imaging Reporting and Data System (BI-RADS) to interpret and report breast imaging findings [4,5]. The purpose of BI-RADS is to ensure consistent reporting and to provide clear management recommendations for patients, with a classification ranging from BI-RADS 1 up to BI-RADS 6. The rankings BI-RADS 1 (negative), 2 (benign) and 3 (probably benign) are generally associated with very low risk of cancer and do not require a follow-up tissue biopsy. Higher rankings, such as BI-RADS 4 (suspicious abnormality) and 5 (highly suggestive of malignancy), strongly recommend a biopsy, while with BI-RADS 6, malignancy is already confirmed via biopsy.

While the BI-RADS system is effective at identifying lesions requiring further investigation, it presents a significant diagnostic dilemma, particularly for lesions classified as BI-RADS 4. This “suspicious” category recommends an invasive tissue biopsy as the standard practice across all its subcategories. However, the BI-RADS 4 category is so broad (with a malignancy risk ranging from just above 2% to as high as 95% [6] across its subcategories 4A, 4B and 4C) that published data show that 70–80% of biopsies performed for these lesions ultimately yield a benign result [7,8,9]. This ambiguous category is also where the BI-RADS system, despite its goal of standardization, is subject to the highest variability in how different radiologists interpret the same mammogram.

Although the BI-RADS 4 subcategories are intended to reflect a gradient of malignancy risk, this category remains a significant diagnostic challenge in clinical practice. Inter-reader variability in the interpretation and classification of imaging findings can lead to inconsistencies in subcategory assignment and subsequent management decisions. As a result, patients are subjected to the anxiety, pain, and potential complications of an invasive biopsy procedure that, in retrospect, could have been avoided. Furthermore, a study demonstrated that women who had a BI-RADS 4 assessment that turned out to be benign (given a subsequent negative biopsy result) are about 10% less likely to return for a subsequent screening [10], demonstrating the potential for a false diagnosis to compromise long-term engagement in recommended cancer monitoring. These downstream effects of false-positive assessments not only places a heavy emotional and physical burden on patients [11] but also impose a substantial economic cost on the healthcare system [12]. There is a clear and urgent need for a non-invasive tool to better stratify risk and reduce the number of unnecessary biopsies.

Our research addresses this challenge by exploring the diagnostic potential of Volatile Organic Compounds (VOCs) in exhaled breath. The distinctive metabolic processes of malignant tumors are known to produce a distinct profile of VOCs, which are released into the bloodstream and subsequently expelled in the breath, creating a unique “breathprint” that can serve as a non-invasive biomarker [13,14,15,16,17,18]. This paper presents a proof-of-concept study using the DiagNoze breathalyzer (Noze Inc., Montreal, QC, Canada) designed to differentiate between malignant and benign pathologies in the challenging BI-RADS 4 cohort. We hypothesize that a predictive model, developed using machine learning on a combination of breath data and BI-RADS classification, could more accurately stratify risk than using BI-RADS assessment alone.

## 2. Materials and Methods

### 2.1. Study Design and Population

#### 2.1.1. Study Design

This study was conducted at the McGill University Health Centre (MUHC) Breast Center, a tertiary referral clinic specializing in breast cancer diagnosis and treatment. The study received full approval from the MUHC Research Ethics Board (REB), and written informed consent was obtained from all participants prior to enrollment. The study was guided by three core design principles: scientific rigor, respect for patient experience, and clinical workflow integration. First, the study was designed to be subtype-agnostic, which included enrolling participants with any form of breast imaging abnormality requiring biopsy, to ensure that the resulting VOC breathprint would be broadly representative and clinically applicable across the disease spectrum. A dedicated breath sample collection room was set up in the Breast Center of MUHC for collecting breath samples from the subtype-agnostic participants. To limit the burden on participants, they were enrolled from the waiting room on the day of their scheduled procedures, such as diagnostic mammogram, ultrasound or biopsy. Further, the breath sample collection was restricted to a single-day procedure, during which the entire sample collection was completed within 1 h–1.5 h, allowing up to 5 samples collected per participant. The ambient humidity and temperature were observed to be <50% RH and <27 °C. Second, stringent inclusion and exclusion criteria were implemented to control for known metabolic confounders, thereby enhancing the specificity of the breath VOC signature. Third, the protocol was designed to align seamlessly with standard clinical pathways, minimizing disruption to care and imposing no additional burden on participants.

#### 2.1.2. Study Population

To ensure the integrity of the breath-based biomarker data, stringent eligibility criteria were applied to control for both internal metabolic and external environmental confounders. Inclusion criteria required participants to be biologically female, between 18 and 80 years of age, recently referred to the clinic for a suspicious breast imaging finding, and capable of providing informed consent. Exclusion criteria were implemented to eliminate factors known to influence breath VOC composition. Individuals were excluded if they had a medical history of asthma, chronic obstructive pulmonary disease (COPD), or diabetes—conditions known to significantly alter endogenous VOC profiles [19,20,21]. Additional exclusions included current smoking [22], consumption of alcohol, tobacco (in a consumable format), vaping, marijuana, or other recreational narcotics eight (8) h prior to the breath test. An additional 1 h fasting limitation that excluded consumption of food, coffee, chewing gum, or consuming any substance other than water was implemented. These measures were essential to ensure that the collected data reflected physiological signals specific to breast pathology, free from confounding metabolic noise.

Eligible participants were recruited from among patients referred to the MUHC Breast Center following abnormal findings on screening mammography. Recruitment occurred during natural waiting periods within the standard diagnostic pathway, such as while awaiting imaging or consultation, allowing for a seamless integration into clinical workflows without disrupting care. Breath sampling was completed in a single visit and required no special preparation or follow-up. The target sample size was 176 participants, reflecting the typical diagnostic distribution observed at the clinic. Group 1 (Controls) included individuals with comprehensive diagnostic evaluation, who were confirmed via biopsy to have benign findings. Group 2 comprised individuals with biopsy-confirmed breast cancer. Importantly, the control group (patients presenting with comparable clinical suspicion but ultimately non-malignant outcomes) provided a highly relevant benchmark for evaluating model performance.

Each participant was asked to provide four to five replicate breath specimens using the DiagNoze breathalyzer (manufactured by Noze Inc., Montreal, QC, Canada), enabling generation of high-resolution VOC breathprint profiles. In parallel, clinical data were extracted from electronic medical records, including diagnostic outcomes from imaging and pathology, tumor characteristics, and relevant genetic information (e.g., BRCA1/2 mutation status).

### 2.2. Device Description

**Figure 1:**
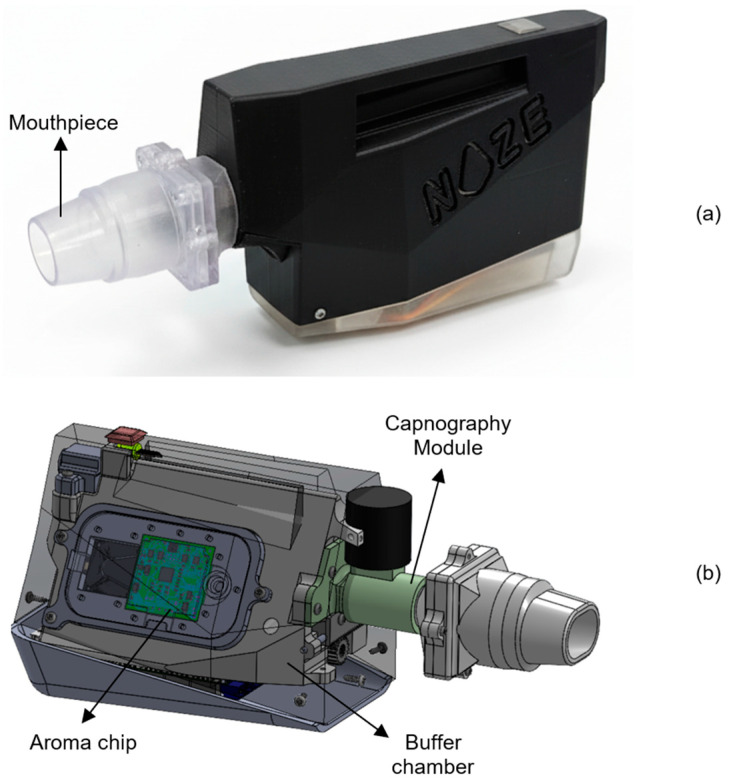
(**a**) The DiagNoze breathalyzer device and its detachable single-use mouthpiece, with (**b**) an internal view of the components: the capnography module, the buffer chamber and the aroma chip module.

Breathprints were recorded from participants using the DiagNoze (manufactured by Noze Inc., Montreal, QC, Canada), an eNose-powered breathalyzer device, as shown in Figure 1a, which digitizes alveolar biomarkers including volatile organic compounds (VOCs) in an exhaled breath specimen. The breathalyzer is composed of two primary components: the mouthpiece and the main unit. The single-patient, disposable mouthpiece streamlines the exhaled breath into the device, filters humidity and is restricted to five exhalation cycles. Each mouthpiece is vacuum packed individually in a clean and sterile environment. The main unit contains three sequential modules: a capnography [23] module, a buffer chamber, and the aroma chip module, as shown in Figure 1b.

To isolate the physiologically relevant portion of the breath, the device has integrated an in-line capnography logic to detect and discard the initial phase of exhalation (commonly referred to as dead space air), which originates from the upper airways and contains minimal metabolic information. Only the alveolar fraction, drawn from the deeper lungs and enriched in endogenous VOCs reflective of systemic metabolism [24], is retained in the subsequent buffer chamber for digitization. This selective sampling process enhances the reliability and biological relevance of the resulting VOC fingerprint.

**Figure 2:**
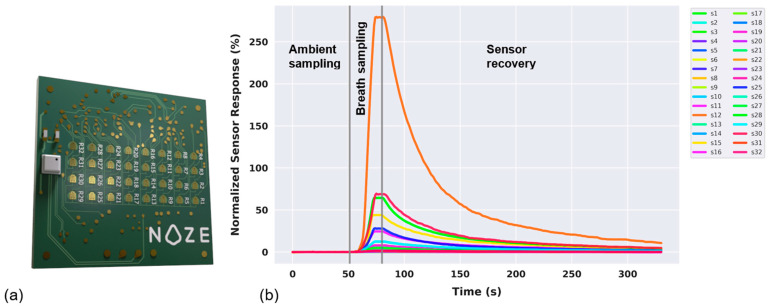
The NOZE Aroma Chip and its corresponding output. (**a**) The aroma sensor chip with its array of 32 sensing elements. (**b**) The time-series plot from the aroma chip during a breath sampling event, showing the ambient sampling, breath sampling, and sensor recovery phases.

The alveolar breath specimen is then transferred from the buffer chamber to the aroma chip [25] (Figure 2a). The aroma chip comprises a cross-reactive chemiresistive sensor array of 32 thin films, each made from a different proprietary polymer-carbon black nanocomposite material [26]. In these thin films, the conductive carbon black network is embedded within chemically diverse polymer matrices, enabling reversible changes in electrical resistance upon exposure to VOCs [27,28]. Differences in polymer chemistry confer varying affinities toward broad classes of VOCs relevant to human metabolism, including aliphatic and aromatic hydrocarbons, alcohols, aldehydes and ketones. Rather than targeting individual molecules, the array is designed to generate overlapping yet distinct response patterns across the thin films, allowing discrimination of complex biological mixtures. This cross-reactive sensing strategy is well suited to capturing disease-associated metabolic signatures, including those linked to oxidative stress, lipid peroxidation, and altered cellular metabolism in cancer [14,15].

As VOCs in the breath interact with the sensor surfaces, they induce specific changes in electrical impedance, which are captured in real time. The result is a high-dimensional, time-resolved dataset (“digital breathprint”) captured at a frequency of 1 Hz across all sensing elements simultaneously (Figure 2b). This dynamic output reflects the full course of the breath-sensor interaction, including ambient-sampling, breath-sampling, and sensor-recovery phases. The multidimensional nature of this data provides a rich foundation for machine learning-based pattern recognition and classification. The DiagNoze breathalyzer and the embedded aroma chip module have been applied and tested in other studies. The performance of the DiagNoze breathalyzer in a peppermint breath study, based on benchmarking protocol for breath analysis developed by Henderson et al., has been reported [25,29] where the benchmark was done against a GC-MS system. Also, a ketone breath analysis study performance with DiagNoze breathalyzer has been reported where the results were benchmarked against a Biosense ketone breath analyzer (Readout Inc., St. Louis, MO, USA) [25].

### 2.3. Breath Sampling Protocol

**Figure 3:**
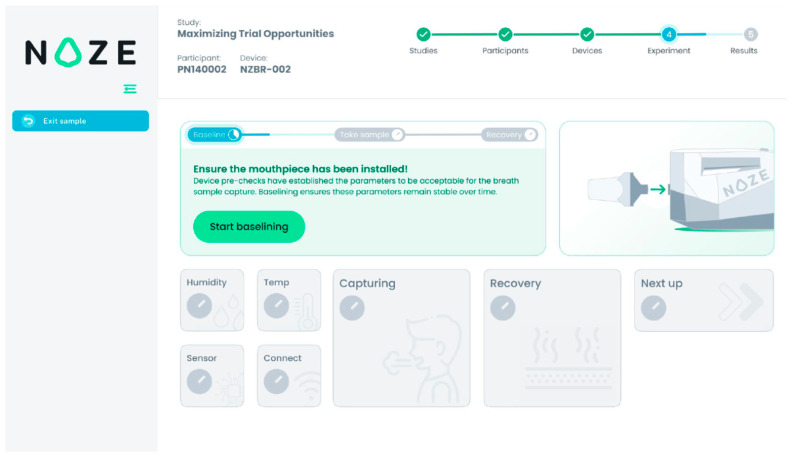
The DiagNoze web-based UI. The UI provides step-by-step guidance for the coordinator, showing the current stage of the breath sampling protocol and real-time quality control checks.

To ensure reproducibility and data integrity, breath collection was conducted using a standardized, operator-guided protocol via the DiagNoze web-based user interface (UI) (Figure 3). Each participant was asked to provide four to five replicate breath specimens to account for natural physiological variability in the breath composition [25].

For each replicate, the UI guided the clinical coordinator through a structured, three-phase measurement cycle:

- Ambient Sampling Phase (30 s): The device initially sampled ambient air, through the mouthpiece, to establish a stable response with respect to the ambient. This step calibrates the sensor array to the room’s background VOC composition, ensuring accurate differential detection during breath sampling. In Figure 3, this phase is referred to as “Baselining”.
- Breath Sampling Phase (5–15 s): With the participant’s nose gently occluded to prevent nasal breathing, a single full exhalation was performed into the mouthpiece. The integrated capnography [23] module automatically identifies the end-tidal (alveolar) portion of the breath and triggers its capture in the buffer chamber. In Figure 3, this phase is referred to as “Capturing”.
- Sensor Recovery Phase (250 s): Following sample capture, ambient air was drawn through the system to facilitate desorption of VOCs from the sensor surfaces, allowing the array to return to the ambient state in preparation for the next measurement. In Figure 3, this phase is referred to as “Recovery”.

Between adjacent specimens, a high-flow purge fan actively evacuated residual VOCs and moisture from the internal components, preventing signal carryover and ensuring full sensor recovery to its equilibrium with the ambient air as the reference state prior to the next measurement. This automated cleaning process preserved the independence and integrity of each breathprint. The entire sampling workflow—including participant instructions, real-time monitoring, and device readiness—was orchestrated through the DiagNoze UI, streamlining operations and optimizing data quality across all replicates. All breathprint data were anonymized at the point of collection and transmitted securely to a managed-access cloud platform for analysis, in compliance with institutional data governance policies. Detailed security protocols are described in the Appendix A.

To assess the reproducibility of measurements across different analytical sessions, we conducted a statistical analysis of the aroma chip data over time. The collected breathprints were chronologically divided into four subsets. For each breathprint, the extremum of normalized responses was calculated, and the distribution of these measurements was analyzed for shifts using *t*-tests. The results indicated no significant shifts (*p*-value > 0.05) between any of the consecutive subset pairs.

### 2.4. Data Preprocessing and Model Building

Developing a malignancy classifier model capable of interpreting complex breathprint data requires a structured, multi-phase approach. The methodology comprised three main stages: (1) preprocessing the raw sensor data into a standardized analytical format; (2) training a machine learning model optimized for clinical relevance; and (3) evaluating model performance using a rigorous, multilayered validation framework.

Breathprints were included in the model development, blind to patient information and biopsy outcome, if they passed the following criteria: (i) ensuring stable sensor responses during ambient sampling, with changes less than 0.01%, (ii) successful breath sample collection confirmed by statistical analysis of sensor response during the breath sampling interval, (iii) validating no data loss during digitization, and (iv) verifying that the device’s mechanism worked as expected for sensor recovery by validating that the most-responsive sensing elements recovering to less than 20% of their peak response.

#### 2.4.1. Data Preprocessing

To prepare the data for building an AI model, we applied a standardized preprocessing protocol involving time-series normalization with respect to the ambient air to account for environmental variability following [30]. The dataset is then partitioned using stratification into multiple folds in order to enable a nested cross-validation [31] to evaluate the model development process. To preserve clinical representativeness and avoid sampling bias, the folds are stratified by diagnostic category, including intermediate BI-RADS 4 subgroups, ensuring consistent outcome distribution across both sets.

#### 2.4.2. Model Architecture and Clinically Optimized Training

**Figure 4:**
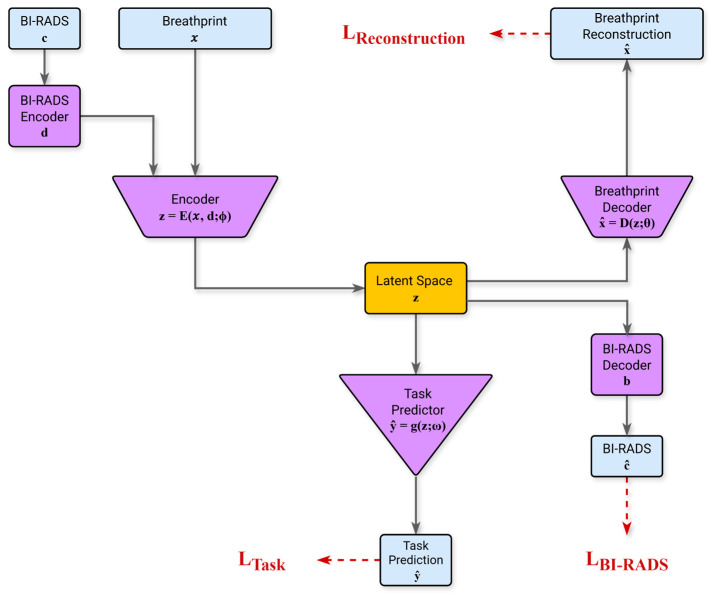
The Model Architecture that takes the BI-RADS category (c) and breath data (x) as inputs. Two encoders map these into a latent vector (z). From this latent vector, a task predictor predicts malignancy (ŷ), while two decoders reconstruct the original breath data ($\hat{x}$) and BI-RADS score ($\hat{c}$).

To learn the key attributes (features) of the digital breathprint that jointly represent its time-series dynamics, we engineered a semi-supervised model based on the Autoencoder (AE) architecture [32]. The Autoencoder architecture facilitates the extraction of key features by compressing the information content of the data into a condensed vector representation within its latent space, Z. The modified architecture shown in Figure 4 incorporates encoders designed to jointly map the breathprint time-series (d) and the associated BI-RADS score (c) into a shared latent vector (z). The encoders/decoders are learned to ensure the latent representation encapsulates the necessary information by reconstructing both the breathprint and the BI-RADS score from (z). Concurrently, a multi-layer perceptron block optimizes the latent space Z, suited most for the malignancy classification task in a supervised fashion.

The model is optimized using a composite loss function comprising L_Task_, L_Breathprint_, and L_BI-RADS_, respectively, representing:

- L_Task_: the error for performing the malignancy classification task
- L_Breathprint_: the error for decoding the breathprint from the latent vector
- L_BI-RAD_: the error for decoding the BI-RADS score from the latent vector

To ensure that the model is aligned with clinical priorities, the training process incorporated several safety-focused optimizations. First, a class-weighted loss function addressed the natural imbalance in the dataset, where benign findings were more prevalent than malignant ones. Second, we explicitly prioritized sensitivity by assigning higher penalties to false negatives (missed cancers), thereby reducing the likelihood of underdiagnosis. Third, model selection is based on a custom clinical utility metric that emphasized generalizability and diagnostic robustness over raw accuracy. A full description of the model architecture, loss functions, and training procedures is available in the Appendix.

#### 2.4.3. Model Cross-Validation

To ensure a fair and clinically meaningful assessment of model performance, we implemented a robust evaluation framework emphasizing reproducibility and diagnostic safety. Rather than relying on a single train–test split, we used the nested cross-validation [31] framework to evaluate the model across multiple partitions of the dataset, reducing the risk of overfitting or optimistic bias. Model predictions were generated using an ensemble approach, where multiple independently trained models contributed to the final output via majority vote. In alignment with our safety-first principle, any vote ties were resolved conservatively in favor of a positive (malignant) classification.

Final performance metrics are reported as the mean and standard deviation across 100 independent runs, providing a stable and reliable estimate of the performance of the generated model following the model development process. During each run, the folds were generated using the original dataset independently. See the Appendix A for more details on the cross-validation structure and the employed ensemble procedure.

## 3. Results

### 3.1. Study Population and Data Distribution

The study enrolled 176 participants. Four to five breath specimen digitization attempts were performed per patient, of which three breathprints were successfully recorded on average across the patients. Fifty-one participants were excluded due to an inconclusive (*n* = 15) or unreported (*n* = 13) BI-RADS score, or having a BI-RADS score not among 3, 4A, 4B, 4C, or 5 (*n* = 6). For 17 participants, sampling the digitized breath aroma failed across all attempts.

The analysis included 125 participants who provided a total of 437 successful breathprints. Of these, 72 participants had confirmed benign findings, contributing 270 successfully recorded breathprints. The remaining 53 participants had biopsy-confirmed breast cancer, from whom 167 breathprints were successfully recorded.

The BI-RADS 4 group (A, B, and C) included 85 participants. Among these, 17 had biopsy-confirmed breast cancer, and 68 had biopsy-confirmed benign findings. A total of 53 and 256 successful breathprints were recorded from these participants, respectively.

Table 1 summarizes the distribution of these 309 successful breathprints.

### 3.2. Predictive Performance in the BI-RADS 4 Cohort

**Figure 5:**
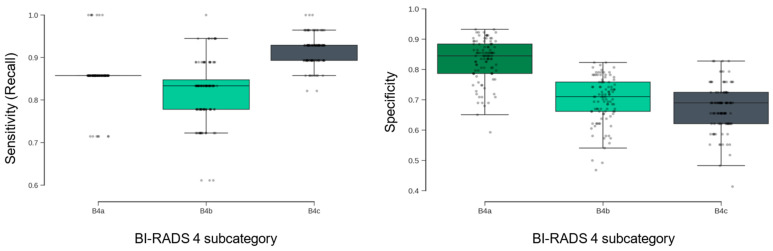
Distribution of model sensitivity (**left**) and specificity (**right**) across 100 repeated cross-validation runs, stratified by BI-RADS 4 subcategory.

The malignancy rate for all BI-RADS 4 cases combined is 17%, meaning most of these cases are benign. To create a model that can meaningfully differentiate between malignant and benign tumors, we included breathprints from patients with BI-RADS scores of 3 and 5 as well. This broader dataset of 437 breathprints was used to train the model, enabling it to learn the data dynamics required for accurate classification. However, the core of this analysis was concentrated on participants whose mammography results were classified as BI-RADS 4 (A, B, and C). These categories represent patients with findings that are considered suspicious. Across the full BI-RADS 4 group, the model achieved a mean sensitivity of 88% ± 3% (Table 2), indicating a high capacity to correctly identify malignant cases. The metric is consistent across BI-RADS 4 subcategories, with particularly high sensitivity maintained in the 4C group. The results are stable across the 100 randomized cross-validation runs. As shown in Figure 5, sensitivity distributions were tightly clustered across subgroups, suggesting minimal variance in model behavior across different partitions of the dataset.

#### 3.2.1. Specificity and Sensitivity Trade-Offs Across Subcategories

Specificity is the ability of a test to correctly identify those without the disease. The model’s specificity demonstrated a decreasing trend from BI-RADS 4A to 4C subgroups (Figure 5, right), with the highest specificity observed in the 4A group. This pattern reflects the model’s sensitivity-focused training protocol, which prioritized the reduction of false negatives in higher-risk categories. While specificity was lower in BI-RADS 4B and 4C, this trade-off was made to preserve high sensitivity in patients with a higher pre-test probability of malignancy.

#### 3.2.2. Summary Metrics and Negative Predictive Value

Table 2 presents comprehensive model performance across all BI-RADS 4 subgroups. The model achieved an overall NPV of 97% ± 1%, indicating a high level of confidence in negative test results. Specificity was maintained at 75% ± 7%, indicating that the model correctly identified approximately three out of four benign cases. This balance between high sensitivity and moderate specificity supports the model’s potential utility as a rule-out tool in diagnostic workflows.

#### 3.2.3. Additional Observations

A strong correlation was observed between mammography lesion size and biopsy outcome, where the median maximum size for malignant cases (21.0 mm) is more than double that of non-malignant cases (10.0 mm), though overlapping minimum sizes suggest size is not the sole diagnostic factor. The patient cohort is skewed towards early-stage disease, with Stage I (45.8%), Stage II (33.3%), and Stage 0, Ductal Carcinoma In Situ (DCIS) (14.6%), while advanced stages are minimally represented. Furthermore, the molecular profile of positive cases is predominantly Luminal A (74.2%), a key finding that guides clinical standard of care, including the application of endocrine or targeted anti-HER2 therapy.

## 4. Discussion

This proof-of-concept study demonstrates that exhaled breath contains a detectable and clinically informative signal capable of distinguishing between malignant and benign findings in women with BI-RADS 4 mammographic assessments. Using a digital olfaction platform and a machine learning model trained with clinically informed constraints, we achieved a mean sensitivity of 88% and an overall NPV of 97%. These results suggest that breath-based diagnostics may offer a promising non-invasive approach for stratifying risk in patients with indeterminate imaging findings.

The clinical implications are particularly relevant for managing BI-RADS 4A and 4B lesions, which account for a high volume of benign biopsies. A high NPV in this setting may enable more conservative management strategies, such as short-interval imaging follow-up, potentially reducing unnecessary procedures and associated patient anxiety. Additionally, according to a previously published study [33], performance remains strong in participants with dense breast tissue, a population in which mammographic sensitivity is known to be reduced. This suggests that a breath-based approach could serve as a complementary modality in cases where traditional imaging has limitations.

The model’s diagnostic behavior was shaped by a deliberate training strategy that prioritized sensitivity. Through the use of class weighting and penalty adjustments, we explicitly reduced the likelihood of false-negative results. This design choice aligns with clinical priorities, particularly in early cancer detection where the cost of a missed diagnosis is high. While this approach resulted in a moderate reduction in specificity, the observed trade-off is clinically appropriate in a rule-out context.

Importantly, the model’s performance was validated using a rigorous cross-validation framework, including 100 independent runs and ensemble-based predictions, ensuring robustness across multiple data partitions. However, the study is limited by its single-center design and the use of a single device platform. Although the results are internally consistent and statistically stable, external validation in multi-center cohorts will be essential to confirm generalizability. These findings support further investigation of breath-based diagnostics in breast cancer. Future studies should aim to evaluate the reproducibility of these results across different clinical settings, devices, and populations, and explore integration with existing diagnostic pathways.

The low positive predictive value (PPV) for categories 4A (28%) and 4B (29%) is largely attributed to the low prevalence of malignancy within these subgroups. For instance, the malignancy rate for BI-RADS 4A is only 6%, indicating that the majority of these cases are benign. This low prevalence inflates the false positive rate, thereby decreasing the PPV, as defined by the formula PPV = TP/(TP + FP). Conversely, the PPV for category 4C (73%) is considerably higher due to a significantly greater malignancy prevalence in that group, which increases the likelihood that a positive prediction is correct.

These results also open the door to a complementary diagnostic paradigm, in which breath analysis is used not as a standalone tool but as a tandem modality alongside mammography and ultrasound. In this scenario, the breath-based test could be administered immediately after a suspicious BI-RADS 4 mammogram, offering an additional layer of risk stratification prior to biopsy. For example, a negative breath test result in a BI-RADS 4A or 4B case, especially given the model’s high negative predictive value, could support a more conservative management approach such as short-term imaging follow-up rather than immediate biopsy. This combinatorial use of imaging and breath diagnostics may help reduce inter-reader variability, alleviate patient anxiety, and optimize clinical decision-making by tailoring biopsy recommendations to a more individualized risk profile.

## 5. Conclusions

This study establishes proof-of-concept that a non-invasive breath test, analyzed using a digital olfaction platform and a clinically optimized machine learning model, can differentiate benign from malignant breast lesions in women with BI-RADS 4 findings. The model achieved high sensitivity (88%) and a negative predictive value of 97%, supporting its potential use as a rule-out tool in the diagnostic workup of suspicious mammograms. Our findings demonstrate that this model achieves high sensitivity and an exceptionally high NPV. These results establish a strong foundation for a tool that could confidently rule out malignancy, potentially sparing a majority of women from an unnecessary biopsy.

By reducing reliance on invasive biopsy in low-risk cases, this technology could alleviate patient burden and streamline clinical decision-making and provide cost savings to an already overburdened healthcare system. The results warrant further validation in larger, multi-center studies to confirm generalizability and evaluate integration into real-world diagnostic workflows.

Despite the good outcomes observed, this study has limitations. The study was designed as a single-center, proof-of-concept with a relatively small sample size, and the performance was evaluated using nested cross-validation within the existing dataset. In the study, we did not evaluate generalizability across different clinical settings or different demographics. Although exclusion criteria were applied to reduce known metabolic confounders, co-morbidities and concomitant conditions may influence breath volatile organic compound profiles and should be more comprehensively evaluated in future studies. Future studies should focus on prospective, multi-center validation in larger and more diverse populations, assessment of inter-site reproducibility, and refinement of system calibration to optimize sensitivity-specificity trade-offs for different clinical use cases. Integration with additional clinical and imaging features may further enhance performance. Successful clinical translation will depend on robust validation, seamless workflow integration, and clear definition of how breath-based testing complements existing diagnostic pathways.

## Figures and Tables

**Table 1:** Participants and Breathprints Distribution.

	Group 1 Benign Lesion	Group 2 Biopsy-Confirmed Breast Cancer	Total
Initial enrolment	110 participants (363 samples)	66 participants (181 samples)	176 participants (544 samples)
Post-exclusion	72 participants (270 samples)	53 participants (167 samples)	125 participants (437 samples)
**BI-RADS Category**			
3	2 participants (7 samples)	0 participant (0 sample)	2 participants (7 samples)
5	2 participants (7 samples)	36 participants (114 samples)	38 participants (121 samples)
4A	26 participants (103 samples)	2 participants (7 samples)	28 participants (110 samples)
4B	34 participants (124 samples)	6 participants (18 samples)	40 participants (142 samples)
4C	8 participants (29 samples)	9 participants (28 samples)	17 participants (57 samples)
4A + 4B + 4C	68 participants (256 samples)	17 participants (53 samples)	85 participants (309 samples)

**Table 2:** Performance metrics including sensitivity, specificity, NPV, and positive predictive value (PPV) across BI-RADS 4 subgroups. Mean and standard deviation are reported across 100 repeated cross-validation runs, stratified by BI-RADS 4 subcategory. The malignancy rate for each category is determined by the ratio of breathprints from patients with biopsy-confirmed breast cancer to the total number of breathprints within that category.

BI-RADS Category	Sensitivity	NPV	Specificity	PPV	Malignancy Rate
4A	86 ± 5%	99 ± 0%	83 ± 7%	28 ± 8%	6%
4B	82 ± 5%	96 ± 1%	70 ± 8%	29 ± 5%	13%
4C	92 ± 4%	90 ± 4%	67 ± 8%	73 ± 4%	49%
4 (A + B + C)	88 ± 3%	97 ± 1%	75 ± 7%	43 ± 6%	17%

## Data Availability

The datasets generated during and/or analyzed during the current study are not publicly available due to the terms of participant consent. Aggregated data supporting the findings are available within the article.

## Appendix A

### Appendix A.1. Secure Data Transmission and Storage

When a breath specimen is successfully digitized into a breathprint, it is securely transmitted from the device to the Noze cloud for analysis. This process is governed by a multi-layered defense strategy designed to protect both the integrity of the data and the privacy of the participant. The first and most fundamental layer of this defense is immediate anonymization. Before any data leaves the device, all personal identifying information is stripped away. Each participant is represented solely by a non-identifiable Personal Unique Identifier (PUI), ensuring that the breathprint is intrinsically unlinked to the individual.

With privacy assured at the source, the anonymized data begins its real-time transfer. This transmission is protected by industry-standard Secure Sockets Layer/Transport Layer Security (SSL/TLS) encryption. This protocol creates a secure, encrypted tunnel between the device and the cloud, safeguarding the data from interception or tampering while in transit.

Upon arrival, the data is housed within a secure, managed-access cloud environment that acts as a digital vault. Here, the data remains encrypted at rest, adding another robust layer of protection. Access to this vault is strictly controlled and requires passing through two distinct security checkpoints: authorized personnel must first connect through a dedicated Virtual Private Network (VPN) and then authenticate with a unique username and password.

### Appendix A.2. Data Preprocessing Details

The full dataset comprised 437 breathprints. Each sample consisted of a multivariate time-series signal acquired from the NOZE aroma chip. The preprocessing pipeline included two key steps:

Time-Series Truncation: Each signal was truncated to a uniform length of 256 s. This window was empirically determined to capture the most information-rich portion of the sensor response, including breath-sampling and sensor-recovery phases, while standardizing input dimensions.

Ambient Normalization: To control for variability in ambient background VOCs, each sample was normalized to its own 30 s ambient-sampling period collected prior to breath-sampling. This allowed the model to focus on biologically relevant signal changes rather than environmental noise.

### Appendix A.3. Model Architecture and Training Strategy

The predictive model employs an autoencoder-based early fusion architecture to integrate multi-modal data [34,35]. This architecture consists of encoder–decoder structures designed to compress and fuse the information content from two distinct modalities: (i) the multivariate breath time series and (ii) the participant’s Breast Imaging-Reporting and Data System (BI-RADS) score. This fusion occurs within a shared latent vector space (Z). The decoders are tasked with reconstructing the original breath signal and the BI-RADS input from the latent vector, thereby ensuring that Z retains the essential features of the input data. Concurrently, a malignancy classifier learns to estimate the likelihood of malignancy using the same latent vector (z). Consequently, the latent space is optimally developed to represent the combined information content of the breathprint and the BI-RADS score in direct relevance to the clinical objective of malignancy prediction.

- The model’s training objective minimizes a combined loss function that includes the standard reconstruction loss (ensuring accurate representation of the breath signal and BI-RADS score) and the classification loss.
- To introduce a crucial clinical bias toward detection, the classification loss employs class-weighted cross-entropy, which selectively up-weights the malignant class during training.
- The models are penalized for overfitting by subtracting the training–validation performance gaps while they are optimized to maximize the F_2_-score, which prioritizes sensitivity (recall) over precision with a ratio of 2.0 for sensitivity and 0.8 for precision, given the dataset skewness towards the biopsy-confirmed benign cases. The coefficients are chosen empirically.

Training is conducted using the AdamW optimizer with a dynamic learning rate scheduler. Regularization strategies included dropout layers and early stopping based on validation loss. This combination of architecture and training logic allowed the model to prioritize clinical safety while maintaining generalizability across a real-world diagnostic population.

### Appendix A.4. Model Parameters

The BI-RADS Encoder, a Multi-Layer Perceptron (MLP), first processes a 5-dimensional one-hot encoded BI-RADS score through a fully connected layer of 5 to 4 neurons with Rectified Linear Unit (ReLU) activation, followed by a second layer of 4 to 2 neurons to create a 2-dimensional vector that is then concatenated with the breathprint as the input to the main Encoder.

The main Encoder includes a series of convolutional blocks, all with kernel size 3, Leaky ReLU activation function, and “Same” padding: Convolutional block 1 uses 1D Convolution to expands its input to 128; block 2 further expands the space from 128 to 256 features, followed by max pooling with stride 2. Block 3 then reduces dimensions from 256 to 128 and block 4 from 128 to 64 attributes. The output is flattened into a 64-dimensional latent vector that is input to the task predictor, the breathprint decoder and the BI-RADS decoder.

The task predictor is implemented as a two-layer MLP utilizing ReLU activation functions. This network is structured with 64 neurons in the first layer and 32 neurons in the second. The output is 2-dimensional, corresponding to the two classes: biopsy-confirmed malignant or benign.

The breathprint decoder uses an upscaling layer to map the latent vector to a feature map with 64 channels and a time dimension equal to half the input sequence length. It then employs a series of 1-dimensional transposed convolutional blocks, starting with block 1 from 64 to 128 channels and block 2 from 128 to 256 channels, each with a kernel size of 3, padding of 1, and Leaky ReLU activation. An unpooling layer performs linear upsampling to restore the original sequence length. block 3 reduces the channels from 256 to 128, and the output Layer performs the final 1-dimensional transposed convolution from 128 to reconstruct the input breathprint.

The BI-RADS decoder is structured as a Multi-Layer Perceptron (MLP). It takes the 64-dimensional latent vector as input. This is then processed through a fully connected block with a ReLU activation function, transitioning from 64 to 4 dimensions and subsequently from 4 to 5 dimensions. The purpose of this architecture is to perform the unsupervised reconstruction of the corresponding 5-dimensional one-hot BI-RADS vector.

The model is trained using the AdamW optimizer with an initial learning rate of 4e^−3^ and a Batch Size of 32, while minimizing a multi-task weighted loss,

L_Total_ = λ_Task_L_Task_ + λ_Breathprint_L_Breathprint_ + λ_BI-RADS_L_BI-RADS_, where λ_Task_, λ_Breathprint_, and λ_BI-RADS_ are empirically set to 1, 1, and 0.5, respectively.

The classification loss, L_Task,_ is calculated using asymmetric weighted binary cross-entropy to dynamically prioritize sensitivity, penalizing false negatives with a weight of 2.0 and false positives with 0.8. An additional base weight is derived from the inverse frequency of the specific BI-RADS subgroup to handle class imbalance.

The reconstruction loss terms, L_Breathprint_ and λ_BI-RADS_, and calculated using the Huber loss with δ equal to 1.

### Appendix A.5. Model Validation Protocol

To obtain an unbiased and robust estimate of generalization performance, a multi-seed, nested cross-validation framework was employed. The full modeling pipeline was repeated 100 times, each with a different random seed to vary the dataset partitioning and model initialization. We employed 7-fold nested cross-validation within each run. This choice was made instead of the more standard 10-fold nested cross-validation due to the limited sample size within specific classes and subgroups, as detailed in Table 1, which necessitated the use of seven folds to ensure proper stratification. Specifically, the Biopsy-confirmed Breast Cancer group for BI-RADS 4A had only seven breathprints. This required seven folds to guarantee that each fold contained at least one breathprint from every category/class, allowing each sample to serve in the test set across multiple rotations for a comprehensive performance assessment. To prevent class imbalance and ensure generalizability, this partitioning was stratified by diagnostic category—including benign, malignant, and the three BI-RADS 4 subgroups—maintaining consistent outcome distributions across both subsets. For each test fold, predictions were made using a committee of 6 independently trained models. Final classifications were determined by majority vote across the ensemble. To reflect a safety-oriented diagnostic approach, any tied votes defaulted to a “positive” (malignant) prediction. The final reported performance (e.g., sensitivity, specificity, F2-score) reflects the mean and standard deviation across the 100 complete runs. This approach prioritizes clinical safety and methodological rigor, reducing the likelihood that reported outcomes result from favorable partitioning or statistical noise.

### Appendix A.6. Model Performance Evaluation Metrics

The model functions as a binary classifier for the breathprint of patients with breast tumors, where a positive output corresponds to a malignant tumor and a negative output indicates a benign case. The performance of the model was evaluated using standard metrics derived from the corresponding confusion matrix. The values for the confusion matrix were obtained by comparing the model’s predicted class (argmax of logits) against the ground truth label from the biopsy results.

The model’s correct predictions are True Positive (TP) for accurately identifying malignant cases and True Negative (TN) for accurately identifying benign cases. The prediction errors are False Positive (FP), mistakenly predicting malignant when the ground-truth is benign (Type I Error), and False Negative (FN), mistakenly predicting benign when the true label is malignant (Type II Error).

In the context of clinical utility, four key classification performance metrics are: (i) sensitivity (recall), (ii) specificity, (iii) Positive Predictive Value (PPV, also known as precision), and (iv) Negative Predictive Value (NPV).

Sensitivity measures the proportion of actual malignant cases correctly identified, by TP/(TP + FN), while specificity determines the proportion of actual benign cases correctly identified, with TN/(TN + FP). PPV calculates the accuracy of positive (malignant) predictions via TP/(TP + FP), while NPV evaluates the accuracy of negative (benign) predictions through TN/(TN + FN).
